# Use of Rituximab in a Sjogren’s Disease–Associated Inflammatory Pseudotumor: A Case Report

**DOI:** 10.1155/crrh/9145050

**Published:** 2026-09-25

**Authors:** Vivian Shing, Lewis Wesselius, Melissa Stanton, Staci Beamer, Tanya Rath, Vivek Nagaraja

**Affiliations:** ^1^ Mayo Clinic School of Graduate Medical Education, Mayo Clinic, Scottsdale, Arizona, USA, mayo.edu; ^2^ Department of Medicine, Division of Pulmonary and Sleep Medicine, Mayo Clinic, Scottsdale, Arizona, USA, mayo.edu; ^3^ Department of Laboratory Medicine and Pathology, Mayo Clinic, Phoenix, Arizona, USA, mayo.edu; ^4^ Department of Cardiovascular Surgery, Mayo Clinic, Phoenix, Arizona, USA, mayo.edu; ^5^ Department of Radiology, Division of Neuroradiology, Mayo Clinic, Phoenix, Arizona, USA, mayo.edu; ^6^ Department of Medicine, Division of Rheumatology, Mayo Clinic, Scottsdale, Arizona, USA, mayo.edu

**Keywords:** case report, inflammatory pulmonary pseudotumor, rituximab, Sjogren’s disease

## Abstract

Inflammatory pseudotumors are uncommon but benign tumors that consist of a proliferation of inflammatory and spindle cells, and they are a rare occurrence in Sjogren’s disease (SjD). We report a rare case of a SjD‐associated inflammatory pseudotumor in the pleural space, which caused mass effect–related discomfort in this patient. A 74‐year‐old female patient with an extensive history of SjD presented with chronic neck and back pain and inflammatory arthralgias. Further imaging showed a right‐sided, paraspinal soft‐tissue mass in the pleural space, raising concern for a possible malignancy. The tumor histopathology revealed a blend of spindle cells, inflammatory infiltrate, and fibrous tissue, and she was eventually diagnosed with an inflammatory pseudotumor. She was initially treated with corticosteroids, which shrank the tumor. However, steroid tapering resulted in the reoccurrence of arthralgias. She was then initiated on rituximab, resulting in an excellent clinical outcome and continued shrinkage of the pseudotumor. This case suggests that rituximab is a possible consideration in the treatment of SjD‐related inflammatory pseudotumors and inflammatory arthralgias, especially when continuous corticosteroid therapy is not ideal.

## 1. Background

Sjogren’s disease (SjD) is a systemic autoimmune rheumatic disease that primarily leads to lymphocytic infiltration and destruction of the salivary and lacrimal glands, causing xerostomia and xerophthalmia, in addition to multisystem involvement [1]. Systemic disease activity can be heterogeneous, with musculoskeletal manifestations (arthralgia and arthritis), lung involvement (interstitial lung disease), and vasculitis, amongst others. Patients with SjD are also at an increased risk of non‐Hodgkin’s B‐cell lymphoma, with reported rates four‐ to sevenfold higher than those in the general population [2]. Inflammatory pseudotumors are a rare manifestation of SjD and have been observed only in a limited number of case reports [2–8]. They are benign but resemble malignancies and can cause mass effect symptoms. We report a rare case of a patient with an SjD‐associated inflammatory pseudotumor located in the pleural space who had an excellent outcome with a combination of oral prednisone and steroid‐sparing therapy.

## 2. Case Presentation

A 74‐year‐old female patient with SjD and osteoporosis presented for evaluation of chronic pain in the neck and upper back with radiation in a right intercostal nerve distribution. Twelve years prior, she had been diagnosed with SjD based on the presence of chronic sicca symptoms and positive serological tests (antinuclear antibody, anti‐SSA, anti‐SSB, and anti‐RNP antibodies). Ten years prior, she had a history of a mass in the dorsal thoracic epidural space extending into multiple neuroforamina, greatest at the T4–T5 level, where there was early extension into the right paraspinal soft tissues. Tumor histopathology was not available as this was evaluated at an outside institution, but it was thought to be an inflammatory pseudotumor. She had been treated with mycophenolate and steroid tapers but experienced little benefit, eventually requiring surgical excision.

**FIGURE 1:**
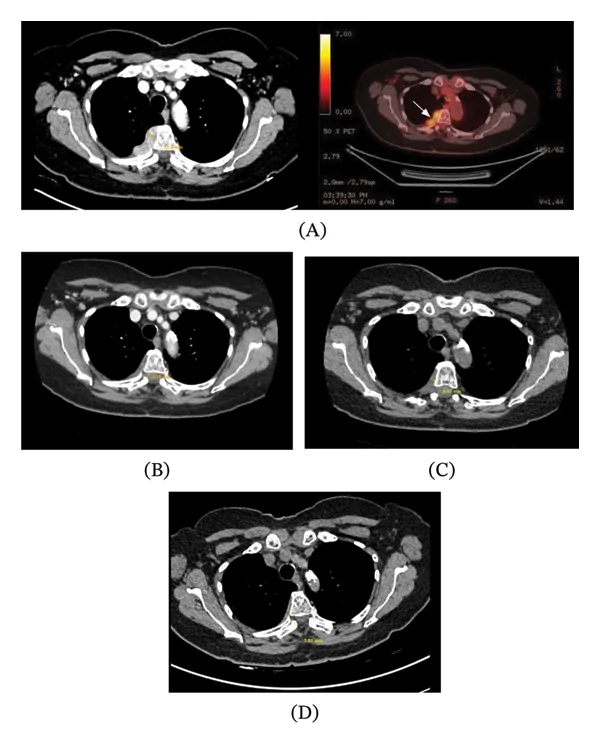
Imaging (axial view) of the chest showing the right‐sided paraspinal mass. (A) CT scan (left) and PET scan (right) at the time of diagnosis. The thickness of the mass measured 12.3 mm, and the corresponding PET scan showed an SUV of 6.5. (B) CT scan 9 months after starting prednisone, showing shrinkage of the mass to 6.15 mm. (C) CT scan 4 months after starting rituximab, showing thickness of the mass to be stable at 6.07 mm. (D) CT scan 3 years after initiation of rituximab infusions every 6 months, showing a reduction in the mass size to 3.82 mm.

For the symptomatology at presentation, a chest X‐ray was obtained, which showed an intrathoracic mass. CT imaging (Figure 1A) showed a right‐sided paraspinal soft‐tissue mass in the pleural space at the T4 level measuring about 12.3 mm in thickness, in the same area as the previously excised tumor. PET scan (Figure 1A) demonstrated hypermetabolic activity within the soft‐tissue mass at the T4–T6 level (SUV: 6.5). Initial considerations included inflammatory pseudotumor, IgG4‐related disease, lymphoma (given her history of SjD), and granulomatous infection—likely histoplasmosis or coccidioidomycosis due to residence in Arizona.

**FIGURE 2:**
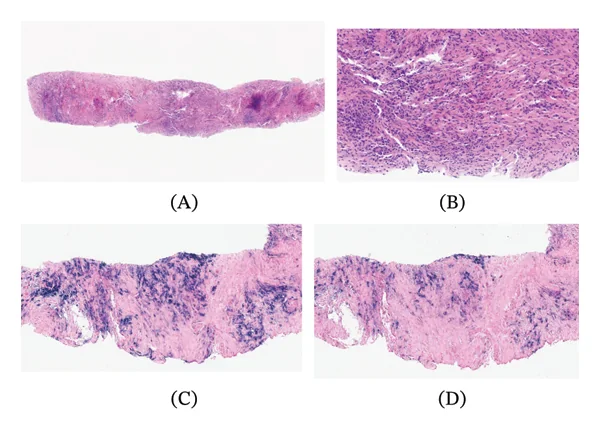
Histopathology of the paraspinal mass. (A) Hematoxylin and eosin staining (4*×*) of the biopsied mass showing spindle cells and chronic inflammatory cells. (B) Hematoxylin and eosin staining (20*×*) of the biopsied mass showing spindle cells and chronic inflammatory cells. (C) Kappa ISH (10*×*) showing polytypic plasma cells. (D) Lambda ISH (10*×*) showing polytypic plasma cells.

The patient underwent CT‐guided biopsy, and histologic sections (Figure 2A,B) showed fibrous tissue with bland spindle cells and a prominent inflammatory background, composed of mostly chronic inflammatory cells—lymphocytes, plasma cells, and histiocytes. No granulomata or neutrophilic inflammation was present. Immunohistochemical stains were performed to further characterize the spindled and inflammatory cells. These showed focal positivity for CD34. IgG4 staining showed an IgG4/IgG ratio of less than 5%, and she had a normal IgG4 serum level, making IgG4‐related disease unlikely. AFB and GMS special stains were negative for acid‐fast bacilli and fungal organisms, respectively. Stains for desmin, SMA, SOX10, ALK1, and pankeratin were negative, which would make a neoplastic process unlikely. Additionally, kappa and lambda in situ hybridization showed polytypic plasma cells, which would be inconsistent with lymphoma (Figure 2C,D). CD3 and CD20 showed a mixed T‐ and B‐lymphocytic component. These histologic findings overall favored the diagnosis of inflammatory pseudotumor, combined with her prior history.

After interdisciplinary discussions, oral glucocorticoid therapy was recommended as an alternative to surgery, as removal of the mass required an extensive chest wall and vertebral body reconstruction. The patient started oral prednisone at a dose of 30 mg per day, and the tumor subsequently decreased in size on imaging to 6.15 mm in thickness (Figure 1B). However, after tapering prednisone, she experienced a recurrence of previous symptoms (neck and upper back pain) and pain and stiffness in the hands, wrists, shoulders, and knees, with a right knee effusion, suggestive of polyarthritis.

**FIGURE 3:**
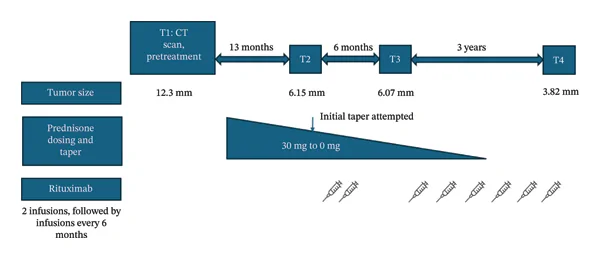
Timeline of the patient’s treatment course, highlighting tumor response to prednisone and rituximab.

Due to coexisting osteoporosis, other steroid‐sparing options had to be considered. Given the predominant lymphocyte and plasma cell population on histopathology of the pseudotumor, B‐cell depletion therapy with rituximab was chosen. She was administered intravenous rituximab 1000 mg, two doses, 15 days apart. Within 1 week of the infusions, she reported experiencing significant improvement in her polyarthritis. At a 6‐month follow‐up appointment, the patient denied any tender or swollen joints, and her patient global assessment (PGA) score decreased from 91 (prior to rituximab therapy) to 22, indicating decreased disease activity. She also had improvement in her chronic back and neck pain symptoms. Initial follow‐up CT imaging a few months after the initiation of rituximab showed no progression of her pseudotumor with residual soft tissue thickening (6.07 mm) (Figure 1C). She continued to receive rituximab infusions every 6 months, and prednisone was completely tapered off following her fourth infusion (Figure 3). CT chest imaging about 3 years following her initial rituximab infusions showed shrinkage of her pseudotumor to about 3.82 mm in thickness (Figure 1D).

## 3. Discussion

Patients with SjD classically manifest with sicca symptoms due to autoimmune‐mediated damage of glandular tissues, although they may also report fatigue, arthralgias, and multisystem involvement. Expression of autoantigens and B‐cell activating cytokines is thought to lead to the hyperactivation and proliferation of B cells in SjD, which can stimulate the formation of ectopic lymphoid structures [9, 10]. These proliferations can potentially transform into malignancies, predisposing patients to lymphomas, most notably non‐Hodgkin’s lymphoma. B‐cell infiltrates can also be seen in IgG4‐related disease, making it an important additional differential to consider. In this patient’s case, biopsy of the mass and histopathology were helpful in further clarifying the diagnosis. The polytypic features of the B cells, as well as a negative IgG4/IgG staining ratio, made lymphoma and IgG4‐related disease unlikely. The presence of bland spindle cells and polymorphic inflammatory cells was the most consistent with an inflammatory pseudotumor. A small number of previous case reports also describe the rare occurrence of Sjogren’s‐associated inflammatory pseudotumors in tissues such as the liver, pancreas, kidneys, and retro‐odontoid region [2–7]. Similar to our patient, these case reports also indicated histopathology findings consistent with chronic inflammation, with the tumors often consisting of lymphocytes and plasma cells.

SjD is primarily managed symptomatically with artificial tears and saliva stimulatory products. While there are no current Food and Drug Administration–approved treatments, disease‐modifying antirheumatic drugs (DMARDs) such as hydroxychloroquine, methotrexate, mycophenolate mofetil, and azathioprine are often used selectively to address systemic manifestations such as arthralgias and fatigue, or organ‐specific manifestations [1]. Previous case reports of Sjogren’s‐associated inflammatory pseudotumors utilized corticosteroids to shrink the mass and nonsteroidal anti‐inflammatory drugs for mass‐related discomfort [2, 6]. In our case, the patient would have reoccurrence of the neck and shoulder pain and exacerbation of arthralgias with tapering of prednisone. Repeated prednisone use was not an ideal therapeutic option due to her osteoporosis.

Rituximab is an anti‐CD20 monoclonal antibody that is usually used to treat lymphoma or as a biologic DMARD for rheumatoid arthritis and other autoimmune diseases. By depleting B lymphocytes, it has been studied as a therapeutic agent for targeting B‐cell hyperactivity in SjD, although randomized controlled trials (RCTs) have not consistently shown efficacy, potentially due to the heterogeneity in the presentation of SjD [11]. A meta‐analysis of available RCTs by Zheng et al. showed that rituximab reduced the serum B cell and IgG levels in SjD patients but did not improve glandular function [12]. In our patient’s case, rituximab was found to be an effective steroid‐sparing treatment in managing her inflammatory arthritis and preventing progression of the inflammatory pseudotumor.

Finally, patients with SjD undergoing consideration for rituximab therapy should undergo screening for hepatitis B and C, human immunodeficiency virus, and tuberculosis, as well as undergo monitoring for hypogammaglobulinemia after infusions. Our patient did not experience recurrent infections requiring hospitalization, and lab monitoring did not show any hypogammaglobulinemia.

## 4. Conclusion

We report a rare case of a pleural inflammatory pseudotumor with a mixture of lymphocyte and plasma cell–rich inflammatory infiltrate along with spindle cells and fibrous tissue in a patient with SjD. Using rituximab, we were able to successfully manage the patient’s inflammatory polyarthritis, reduce the size of her tumor, improve her quality of life in terms of pain and fatigue, and avoid the need for a major surgical procedure. Previous clinical trials suggest that rituximab may benefit specific subsets of SjD patients depending on their clinical presentation and predominant pathophysiological mechanisms. This case suggests that rituximab could be a therapeutic consideration for SjD patients with inflammatory lesions (supported mechanistically by histopathology) or inflammatory arthropathy who are unable to tolerate corticosteroid tapering. However, further research is needed to better define which patients would benefit most from DMARDs or biologics such as rituximab.

**Nomenclature**
Anti‐SSA: Anti–Sjogren’s syndrome–related antigen A
Anti‐SSB: Anti–Sjogren’s syndrome–related antigen B
Anti‐RNP: Antiribonucleoprotein
CT: Computerized tomography
DMARD: Disease‐modifying antirheumatic drug
PET: Positron emission tomography
PGA score: Patient global assessment score
PROMIS CAT: Patient‐Reported Outcomes Measurement Information System Computer Adaptive Test
SjD: Sjogren’s disease

## Funding

No funding was used for this manuscript.

## Ethics Statement

No approval was required by the institution given that this was a case report. Consent for publication was obtained from the patient.

## Consent

Written and signed consent for publication was obtained from the patient.

## Conflicts of Interest

T.R. was the second Past President of the Eastern Neuroradiological Society. The other authors declare no conflicts of interest.

## Supporting Information

Additional supporting information can be found online in the Supporting Information section.

## Supporting information


**Supporting Information** CARE‐checklist‐English‐2013

## Data Availability

Data sharing is not applicable to this article as no datasets were generated or analyzed during the current study.
